# Co-occurrence of transcription and translation gene regulatory features underlies coordinated mRNA and protein synthesis

**DOI:** 10.1186/1471-2164-15-688

**Published:** 2014-08-19

**Authors:** Ana Tamarkin-Ben-Harush, Edna Schechtman, Rivka Dikstein

**Affiliations:** Department of Biological Chemistry, The Weizmann Institute of Science, Rehovot, 76100 Israel; Department of Industrial Engineering and Management, Ben Gurion University of the Negev, Beer Sheva, 84105 Israel

**Keywords:** TATA-box, TATA-less, Transcription, Translation, uAUG, 5′UTR, 3′UTR, uORF

## Abstract

**Background:**

Variability in protein levels is generated through intricate control of the different gene decoding phases. Presently little is known about the links between the various gene expression stages. Here we investigated the relationship between transcription and translation regulatory properties encoded in mammalian genes.

**Results:**

We found that the TATA-box, a core promoter element known to enhance transcriptional output, is associated not only with higher mRNA levels but also with positive translation regulatory features and elevated translation efficiency. Further investigation revealed general association between transcription and translation regulatory trends. Specifically, translation inhibitory features such as the presence of upstream AUG (uAUG) and increased lengths of the 5′UTR, the coding sequence and the 3′UTR, are strongly associated with lower translation as well as lower transcriptional rate.

**Conclusions:**

Our findings reveal that co-occurrence of several gene-encoded transcription and translation regulatory features with the same trend substantially contributes to the final mRNA and protein expression levels and enables their coordination.

**Electronic supplementary material:**

The online version of this article (doi:10.1186/1471-2164-15-688) contains supplementary material, which is available to authorized users.

## Background

Expression of protein-encoding gene in eukaryotes is an intricate process that includes several distinct steps of transcription, mRNA processing and mRNA translation. Each of these stages is controlled by cis-regulatory elements present in the DNA and the mRNA. Transcription is governed by two major types of DNA elements, enhancer and core promoter. Enhancer elements serve as binding sites for transcription regulatory factors and can function independently of their position. Core promoter elements, such as TATA-box and Initiator, are situated around the transcription start site (TSS) and are the sites on which the basal transcription machinery assembles. As such these elements have central role in determining promoter strength [1–3].

Cis-regulatory elements present in the mRNA are central to the control of protein synthesis. Specifically the nucleotide sequence surrounding the initiating AUG [4, 5], the presence of an AUG(s) upstream to the main ORF (uAUGs) [4, 6–8], the lengths of the 5′ and 3′ un-translated regions (UTRs) and the occurrence of stem–loop structures in the 5′UTR [9–14], all influence the rate of protein synthesis. Previous genomic and functional studies suggest that uAUGs act to reduce translation of the downstream ORF either of specific genes or globally [4, 6–8]. The presence of uAUG in eukaryotic mRNAs is highly prevalent, reaching almost half of protein coding genes [6, 15].

Here we investigated the relationship between various regulatory features of transcription and translation encoded in mammalian genes using bioinformatics and functional analyses. Our findings revealed remarkable coupling of several regulatory features that act in the same direction which substantially contribute to mRNA and protein levels and facilitate their coordinated expression.

## Results

### The highly transcribed TATA-box genes have lower frequency of uAUG

**Figure 1:**
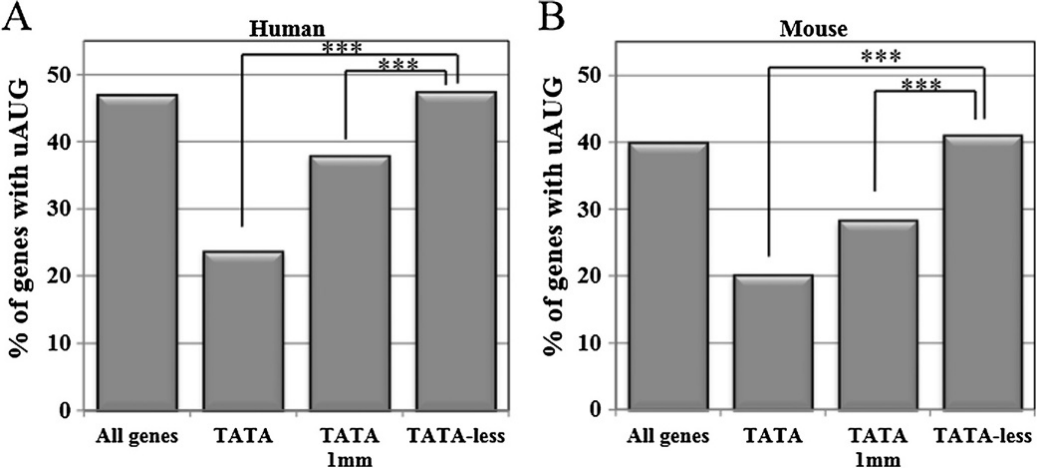
**The prevalence of uAUG in human (A) and mouse (B) genes bearing canonical TATA, TATA with up to one mismatch (1 mm) or TATA-less.** All genes refer to the sum of TATA (with up to one mismatch) and TATA-less genes. ***denotes statistically significant difference *p* < 0.001.

The TATA box is a well-characterized strong core promoter element that is known to be associated with high transcriptional rate [16–21]. Previously we have shown that TATA-containing genes tend to have short length and reduced 5′ and 3′UTR size [20]. However the relationships of these and other features such as uAUGs and coding sequence (CDS) length with translation efficiency were not investigated. To address these issues we identified the TATA-box genes by searching the −40 to −15 region, relative to the annotated TSS of the UCSC database, for the TATAWAG sequence (allowing zero to one mismatch). With this definition of the TATA-box the frequency of this motif is 5% and 8.5% in human and mouse genes, respectively. The same frequency of the TATA-box was found with the DBTSS database, which contains TSSs from CAGE (Cap Analysis of Gene Expression) data. We then compared the frequency of uAUG in all genes to genes containing or lacking TATA-box in their promoter. Consistent with earlier reports [22–24] we found that a considerable fraction of human (~47%) and rodent (~40%) mRNAs possess at least one uAUG in their 5′UTR (Figure 1A and B). Interestingly, in both human and mouse the percentage of uAUG bearing genes is the lowest in the canonical TATA-box group, higher in the one-mismatch TATA-box and the highest in TATA-less group (Figure 1A and B). In other words the frequency of the TATA-box among uAUG genes is lower than uAUG-less genes (3.9% vs. 5.7% in human and 6% vs. 10.2% in mouse, respectively). Thus the prevalence of uAUG negatively correlates with the presence and the strength of the TATA-box. We also carried gene ontology analysis of the uAUG and uAUG-less genes and found some differences with enrichment of several functional categories (Additional file 1: Table S1).

### TATA-box genes lacking uAUG are associated with positive translation regulatory features and higher translation

**Figure 2:**
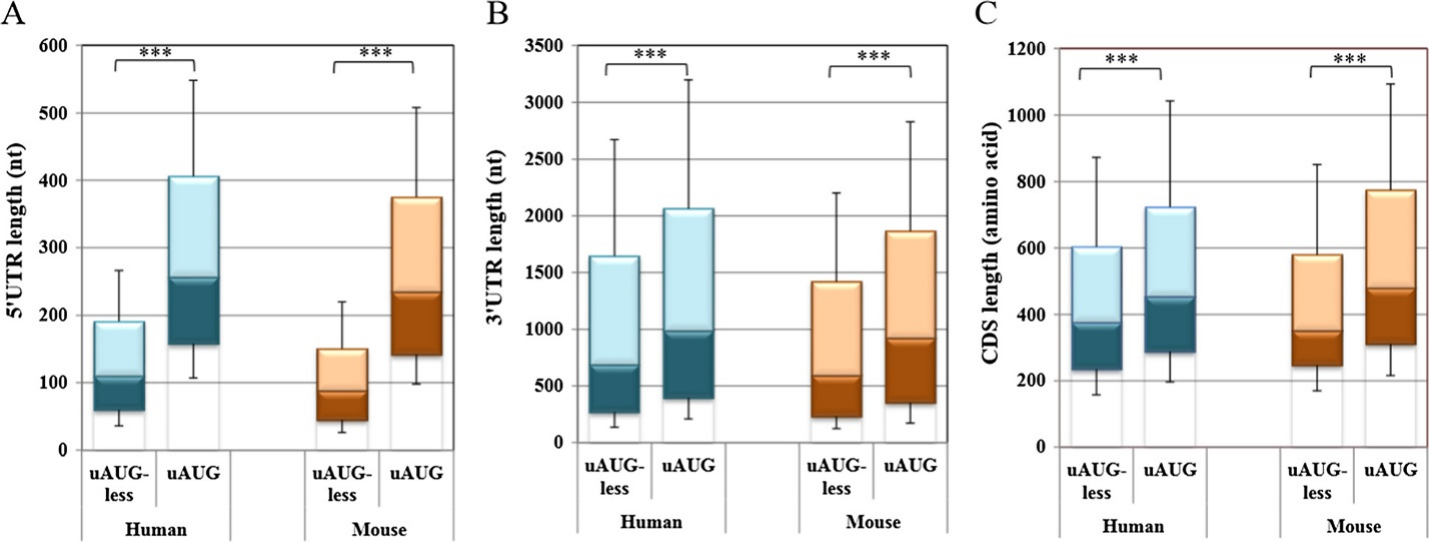
**Translational regulatory features among genes bearing or lacking uAUG. A-C**. Human and mouse uAUG-less and uAUG genes were analyzed for the length of their 5′UTR **(A)**, 3′UTR **(B)** and coding region **(C)**. The data is presented as boxplots with the median, 25% and 75% quartile values; the top and the bottom whiskers represent the 75–87.5% and 12.5-25% of the population, respectively. In all figures the differences were calculated using either Mann Whitney or Kruskal-Wallis test and *, **and ***denote *p*-value < 0.05, 0.01, 0.001, respectively. The blue and the brown colors represent human and mouse data, respectively.

To examine further the relationship between various translation regulatory features human and mouse genes were first grouped as either lacking or containing uAUG (uAUG-less and uAUG, respectively). As shown in Figure 2, remarkable differences exist between the two gene sets both in human and in mouse. The 5′UTR of uAUG-less genes is substantially shorter than that of uAUG genes (Figure 2A). This pattern was repeated with the 3′UTR length and the ORF (CDS) length: uAUG-less genes tend to have significantly shorter 3′UTR and ORF than uAUG genes (Figure 2B and C). While the length of the 5′UTR may be linked to the presence of uAUG, the lengths of the 3′UTR and the CDS have no apparent natural connection to the presence or absence of uAUG in the 5′UTR, yet these translation regulatory traits tend to cluster on mRNAs.

**Figure 3:**
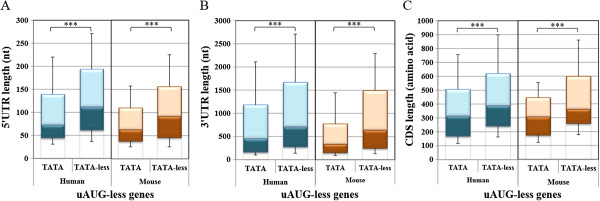
**TATA-box genes lacking uAUG are associated with better translation regulatory features. A-C**. TATA (with up to one mismatch) and TATA-less human and mouse genes, lacking uAUG in their 5′UTR, were analyzed for the length of their 5′UTR **(A)**, 3′UTR **(B)** and coding region **(C)**. The analysis of TATA and TATA-less genes bearing uAUG in their 5′UTR is shown in (Additional file 1: Figure S1). The blue and the brown colors represent human and mouse data, respectively.

Next we compared the translation regulatory features between the TATA (with up to one mismatch) and the TATA-less groups, each divided into uAUG and uAUG-less subsets. We found dramatic differences in all parameters among the uAUG-less subsets, in both human and mouse (Figure 3). Specifically, the 5′UTR, the 3′UTR and the ORF lengths were significantly shorter in the TATA than in TATA-less genes. However in the uAUG containing genes the differences between TATA and TATA-less are much smaller (Additional file 1: Figure S1). These findings are consistent with those reported previously [20] but the present analysis revealed that these differences exist primarily among the uAUG-less subsets. Thus the TATA-box genes that lack uAUG are associated with additional positive translation regulatory features.

**Figure 4:**
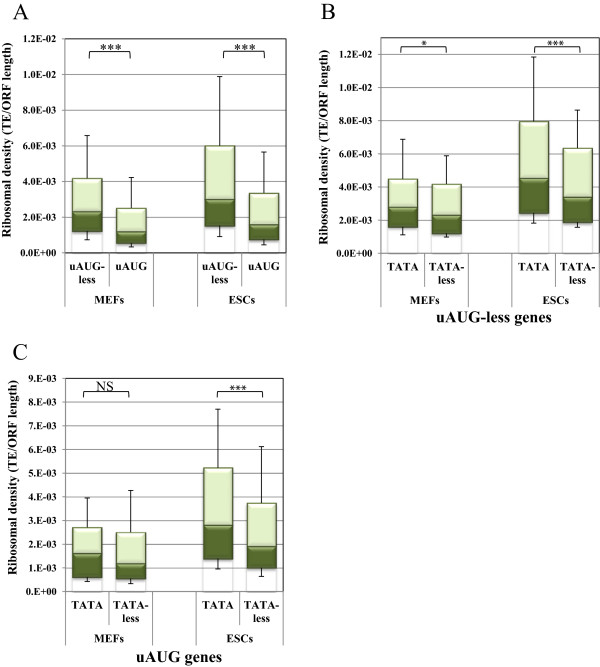
**TATA-box genes are associated with higher translational activity. A**. The translational activity of mouse genes lacking (uAUG-less) or bearing uAUG (uAUG) is presented as ribosomal density values which were derived from two global ribosomal profiling experiments in MEFs and mESC [25, 26] and presented as boxplots. **B**. Ribosomal density values of TATA (with up to one mismatch) and TATA-less mouse uAUG-less genes from the ribosomal profiling data described above. **C**. Ribosomal density values of TATA and TATA-less mouse genes bearing uAUG. The green color of the bar represent the data taken from mouse ribosomal profiling studies [25, 26].

To test the relationship between regulatory features of genes and protein synthesis we retrieved genome-wide translation efficiency data from two recent ribosome-profiling studies from mouse cells. The first contained data of 4,840 genes from mouse embryonic fibroblasts (MEFs) [25] and the second of 10,220 genes from mouse embryonic stem cells (mESCs) [26]. The relationship between 5′UTR, 3′UTRs and the CDS lengths with translation efficiency was assessed using a Spearman rank correlation coefficient. The results revealed a moderate but significant negative correlation between 5′UTR (−0.226, p < 0.0001) and 3′UTR (−0.429, p < 0.0001) lengths and translation efficiency (TE = ribosome reads/total mRNA). The negative correlation between ORF length and translation efficiency was very small (−0.058, p < 0.0001) and may be explained by the RNA-seq methodology used in these studies. For the analysis of the translational activity we calculated the ribosomal density of each gene, which is the ratio between the TE of each transcript and the length of the coding sequence (TE/CDS length). Assessment of ribosomal density of uAUG-less and uAUG in MEFs and in mESCs revealed that the uAUG-less genes show significantly greater ribosomal density than uAUG genes (Figure 4A), which is in agreement with the notion that uAUG attenuates translation from the major ORF.

Considering that TATA genes are associated with positive translational features we would expect these highly transcribed genes to be efficiently translated. Therefore the two datasets were used to compare the translation levels of TATA and TATA-less genes, containing or lacking uAUG. We observed that among the uAUG-less genes, the TATA set showed significantly higher ribosomal density levels than that of the TATA-less set both in MEFs and mESCs (Figure 4B). While with the MEFs no significant differences were seen between the uAUG genes, with the ESCs the ribosomal density of the TATA set was higher (Figure 4C). Together, the analysis of the regulatory features and translational activities support the notion that regulatory traits in transcription and translation were evolved to act in a similar trend.

### Co-occurrence of translation and transcription regulatory trends

**Figure 5:**
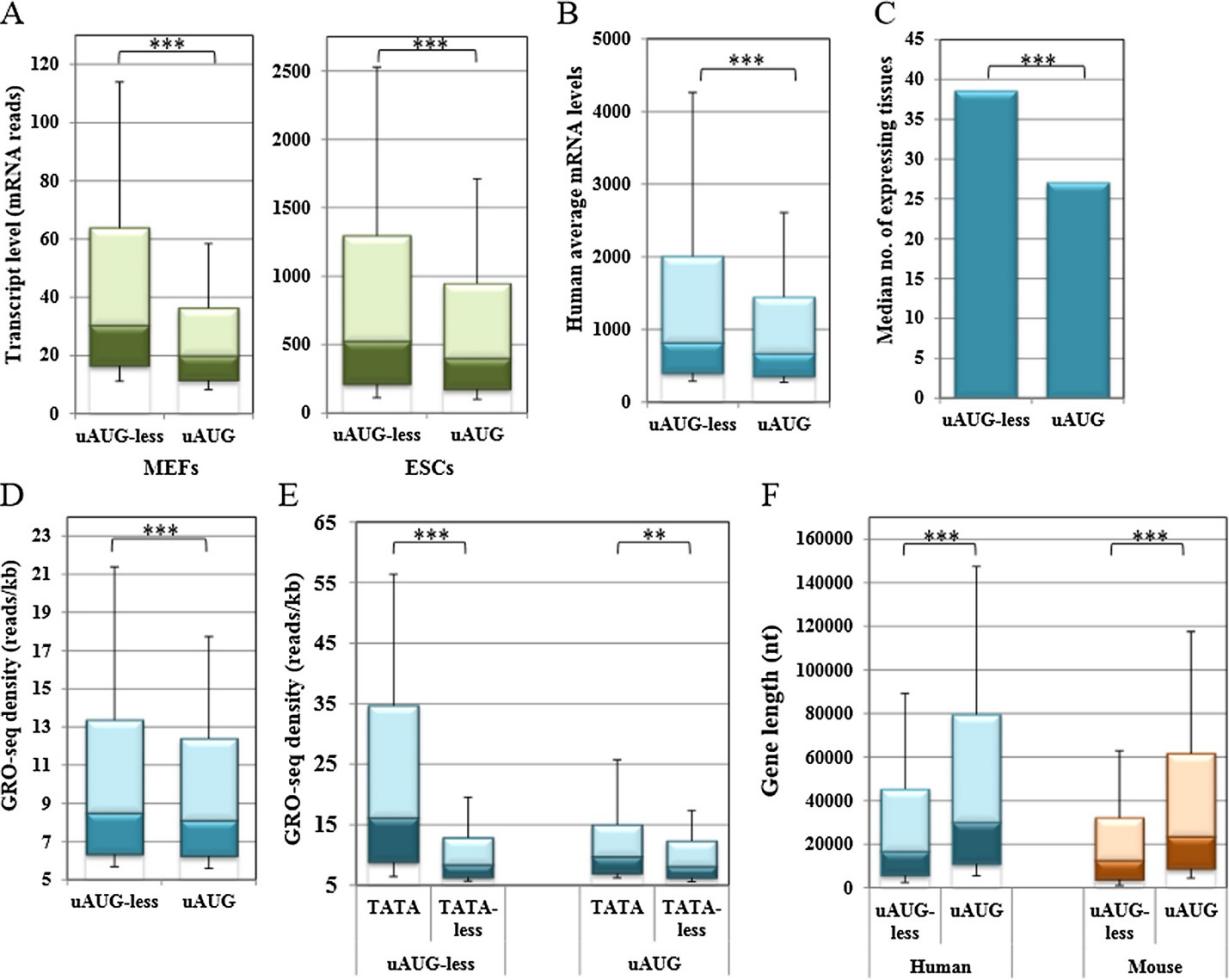
**Relationship between uAUG, mRNA levels, transcription and gene length. A**. Boxplot presentations of transcript levels retrieved from MEFs and mESC ribosomal profiling data [25, 26], in genes without and with uAUG. **B**. A boxplot presenting the average mRNA levels of human uAUG-less and uAUG genes, retrieved from the SymAtlas v1.2.3. **C**. A graph presenting the median number of tissues in which each gene in the uAUG-less and uAUG sets is expressed. The data was retrieved from the SymAtlas v1.2.3. **D**. A boxplot presenting the transcriptional activities of uAUG-less and uAUG genes, which were retrieved from Global nuclear Run-On experiment (Gro-Seq) [29]. **E**. A boxplot presenting the transcriptional activities derived from the Gro-Seq data of TATA and TATA-less genes, divided according to the presence or absence of uAUG. **F**. Boxplots presenting the overall gene length of uAUG-less and uAUG human and mouse genes. The blue and the brown bars represent human and mouse data, respectively. The green bars represent the data taken from mouse ribosomal profiling studies [25, 26].

As a positive transcription regulatory element such as TATA-box was found to be associated with positive translation regulatory features we were prompted to examine general links between transcription and translation. We first analyzed the relationship between ribosomal density and the mRNA levels by Spearman rank correlation coefficient analysis, using the data retrieved from the ribosomal profiling experiment described above [25, 26]. Interestingly, significant positive correlation of 0.418 (p < 0.0001) was found between ribosomal density and mRNA levels. As ribosomal density, represents the efficiency by which each mRNA molecule is translated, independently of the number of RNA molecules, this correlation is unexpected and is not the same as the correlation between mRNA abundance and protein abundance reported previously [27, 28]. To gain further insight into the underlying basis of this connection we compared the transcript levels between the uAUG-less and uAUG gene sets. Remarkably, uAUG-less genes, which are translated more efficiently, tend to have significantly higher mRNA reads in both MEFs and mESCs measurements (Figure 5A). Likewise we found negative correlations between mRNA levels and translation features such as 5′UTR (−0.2, p < 0.0001), ORF length (−0.461, p < 0.0001) and 3′UTR length (−0.368, p < 0.0001).

To examine further the relationship between mRNA levels and translational features observed for mouse genes, we similarly analyzed human gene expression data that was downloaded from the gene expression atlas SymAtlas v1.2.3. This database contains expression data of thousands of human genes from 79 tissues and cell types. We determined the average expression of each gene in all tissues, setting a threshold of 200, a value that is above background. Then we determined the distribution of the average expression of each gene in uAUG-less and uAUG sets using boxplots. Here again it appears that human uAUG-less genes tend to have significantly higher levels of mRNA than uAUG genes (Figure 5B). This is particularly highlighted in the upper 50% of the gene population that is distributed more towards the higher expression levels, both in the human and the mouse data (Figure 5A and B). A similar pattern is observed when the maximal, rather than the average expression of genes is analyzed (Additional file 1: Figure S2). The number of tissues in which each gene is expressed was also determined in the two gene sets, and we found that uAUG-less genes tend to be expressed in more tissues than uAUG genes (Figure 5C).

The analysis shown in Figure 5A and B is derived from steady state mRNA levels data. To examine whether the transcription process directly contributes to the differences seen in mRNA levels between uAUG-less and uAUG gene we retrieved RNA-seq data from a global Nuclear Run-On experiment (Gro-seq), which directly measures the level of ongoing transcription for all genes [29]. The same general trend was found, as uAUG-less genes display higher levels of transcription than those with uAUG (Figure 5D). These findings support the idea that uAUG genes are less efficiently transcribed than uAUG-less genes. It has been previously suggested that uAUG is associated with a shorter mRNA half-life [23], therefore it can be presumed that the combined effects of lower transcription and elevated decay rates are responsible for the marked difference in the steady state mRNA levels (Figure 5A and B). We also analyzed the transcription efficiency of TATA and TATA-less genes divided into uAUG-less and uAUG subsets. The results revealed the expected differences between TATA and TATA-less genes but remarkably this difference is much less dramatic among the uAUG genes (Figure 5E).

An important parameter that is known to influence transcription efficiency is the gene length [20, 30]. Upon analysis we found substantial differences in gene length between uAUG-less and uAUG genes in human and in mouse (Figure 5F), the median gene length in uAUG-less genes being almost half of that in uAUG genes. Exon count analysis showed the same trend (Additional file 1: Figure S3).

Next, expression data from multiple tissues of 6804 human genes were divided with into top 25% and bottom 75% expressed genes and determined the percentage of uAUG bearing genes in the two groups and found that the top 25% gene set has lower uAUG genes than the bottom 75% set (Additional file 1: Figure S4A). The prevalence of uAUG in the top 10% expressed genes is even lower. A similar trend was observed with the GRO-seq data (data not shown). To examine whether it is just the presence of the uAUG that is associated with the reduced mRNA levels we compared the translational regulatory features of the top 25% and bottom 75% expressed genes (at the mRNA level) within the same class, either uAUG-less or uAUG (Additional file 1: Figure S4B-E). While no significant difference is observed in the 5′UTR length between the high and the low expressing genes, clear and marked differences are seen with the lengths of 3′UTR, CDS and overall gene size, these features being much shorter in the higher expressing set both in uAUG-less and uAUG groups. These findings clearly show that while uAUG and the 5′UTR length are important, they are insufficient to account for the association between translational and transcriptional features reinforcing that the co-occurrence of other features also contribute to final expression levels.

## Discussion and conclusions

**Figure 6:**
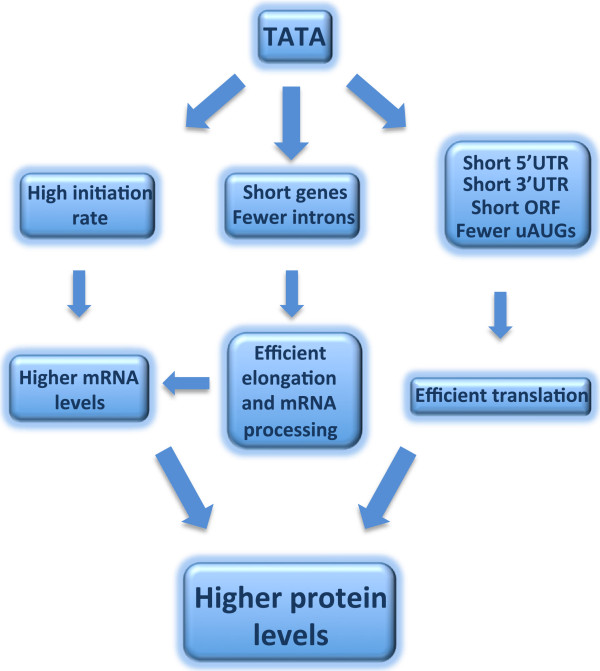
**A scheme demonstrating how coordination of transcription and translation rates is achieved in the TATA-box gene set, by coupling multiple transcription and translation regulatory features that have similar trend.**

The present study demonstrates that various transcription and translation regulatory features were co-evolved in the same direction. Specifically we observed that translation regulatory features acting positively or negatively are linked to transcriptional control features, such as TATA-box and gene length, that function in the corresponding direction. Our findings suggest that clustering of various structural as well as regulatory features, which have the same trend but at different stages of gene expression, can be regarded as a powerful and general mechanism for coordinating the various gene expression stages. This coordination is particularly apparent in the TATA-box gene set as illustrated in Figure 6. In transcription, the TATA-box acts by increasing the rate of initiation [19, 31, 32]. TATA-box genes are also very short and have fewer introns [20] therefore their transcription elongation and mRNA processing is more efficient. The combined effects of these features give rise to high levels of mRNAs [20]. On the translation side, the TATA-box gene set is also characterized by shorter 5′ and 3′ UTRs, smaller ORF size and lower incidence of uAUG. Consistent with that we show that the mRNA molecules generated from these genes tend to be more efficiently translated. Thus it appears that the coupling of all these transcription and translation regulatory features results in much higher level of protein production. An exception is the fraction of TATA genes that also have uAUG. In these genes the advantage of the TATA box in transcription seem to be lost and is accompanied with high prevalence of translation inhibitory features and lower translation.

The link between transcriptional and translational features is not limited to the TATA gene class as analysis of structural organization and functional features of mammalian genes bearing or lacking uAUG, revealed close association with mRNA levels and transcriptional rate as well as other translation regulatory traits that display the same trend. The negative correlation between mRNA abundance and uAUG has been previously noted [23, 27] but this was mostly attributed to the reduction in steady-state mRNA levels and mRNA half-life. The analysis of the Gro-Seq data which reflects only mRNA synthesis rate, revealed that the lower mRNA level associated with uAUG is a combination of lower synthesis with lower stability.

Several recent studies uncovered the associations between features present in the mRNA and protein abundance in yeast and mammalian cells (for example see [27, 28, 33]). These studies also reported that mRNA features, such as lengths of the 5′UTR, 3′UTR and CDS as well as uORF are associated with each other as we also find here. Our analysis further extends these findings by demonstrating that the translational activity of each mRNA is correlated with mRNA abundance. Furthermore, we show that 5′UTR, 3′UTR and CDS as well as uORF are also associated with genomic features that influence the rate of mRNA synthesis, in particular promoter features (TATA vs. TATA-less) and gene length.

A major biological implication resulting from our findings is the ability of the eukaryotic cell to synchronize, to some extent, the transcription and translation rates, through various regulatory features operating in the same direction. The gene ontology analysis (Additional file 1: Table S1) which revealed enrichment of functional categories associated with uAUG and uAUG-less genes, provides examples for coordination of expression level with biological activity. For instance, transcription factors are known to be transcribed at low levels and indeed these factors have higher prevalence of uAUG. On the other hand structural components such as nucleosomal proteins that are highly expressed at the mRNA and protein levels tend to lack uAUG.

In summary, the analysis of transcription and translation data reported here revealed significant association between mRNA levels that reflect transcriptional activity and decay, and translation efficiency.

## Methods

### Selection of genes and analyzing their features

Gene data and sequences were retrieved from UCSC Genome Browser website (http://genome.ucsc.edu/) in which the Feb. 2009 assembly was used for human genes and July 2007 assembly for mouse genes. Using RefSeq track in ‘Table browser’ we downloaded the desired genomic sequence output (CDS and UTRs) for the different groups of genes. To identify uAUG-bearing genes the 5′UTR sequence of all genes were retrieved and analyzed by a PERL code designed to identify the ATG triplet. Genes were subsequently divided into two groups: with and without AUG triplet codon in their 5′UTR (uAUG and uAUG-less, respectively). Sequences of the gene set of interest were analyzed in Galaxy (https://usegalaxy.org/root), a web-based platform for data managing [34–36] and, using EMBOSS ‘infoseq’ tool the length of the sequences was retrieved. Gene length was calculated using the difference between transcription start and end positions in ‘selected fields’ output format. The number of exons was retrieved from exonCount field. Ribosomal density was determined as follows:


Classification of genes according to their function was done using the gene-annotation enrichment analysis (http://david.abcc.ncifcrf.gov/).

### Identification of TATA-box bearing genes

The nucleotides sequences from −40 to −15 upstream to the UCSC TSS, were retrieved. Using the ‘pattern matching’ tool in Regulatory Sequence Analysis Tools (RSAT) site (http://rsat.ulb.ac.be/) we searched for the TATA-box sequence of TATAWAG (allowing zero to one mismatch) in this region. The RefSeq output was transformed into official gene name to remove duplicates.

### Gene expression

Gene expression data was analyzed as previously described [20]. The transcription Gro-Seq data was retrieved from Core et al., [29]. To avoid bias generated by proximal promoter pausing the reads of the first 1 kb were avoided. The number of reads divided by the gene length (minus 1 kb), reflected the transcription level of each gene. A ratio below 5 reads/kb was considered background. For mouse mRNA levels the total RNA-seq data derived from the ribosomal profiling studies [25, 26] were used.

### Statistical analyses of gene features

The distributions of the gene features are skewed therefore non-parametric procedures were used to compare between the groups features, Mann–Whitney U-test for two and Kruskal-Wallis test for more than two samples. The differences between uAUG prevalence among the groups was analyzed by chi-square test and the Spearman’s rank correlation coefficient analysis between MEF’s translation efficiencies and the length of mRNA features was performed using the STATSTICA® software.

## Electronic supplementary material

Additional file 1: Figure S1: Translational regulatory features among TATA and TATA-less genes bearing uAUG. A-C. Human and mouse genes containing uAUG in their 5′UTR were grouped according to the presence and absence of a TATA-box in their core promoter region and analyzed for the length of their 5′UTR (A), 3′UTR (B) and coding region (C). The data is presented as boxplots with the median, 25% and 75% quartile values; the top and the bottom whiskers represent the 75–87.5% and 12.5-25% of the population, respectively. In all figures the differences were calculated using the Kruskal-Wallis test and * and *** denote *p*-value < 0.05 and 0.001, respectively. NS, statistically non significant. The blue and the brown bars represent human and mouse data, respectively. **Figure S2:** A boxplot presenting the maximal mRNA levels of human uORF-less and uORF genes, retrieved from the SymAtlas v1.2.3. **Figure S3:** Boxplots presenting the number of exons in human and mouse uORF-less and uORF genes. The blue and the brown bars represent human and mouse data, respectively. **Figure S4:** Highly mRNA expressing genes are associated with better translational features. A. The prevalence of uAUG in the top 10%, 25% and the bottom 75% mRNA expressing genes. The differences are statistically significant p < 10^−4^. B-E. Top 25% and bottom 75% mRNA expressing genes, containing or lacking uAUG were analyzed for the length of their 5′UTR (B), 3′UTR (C), coding region (D) and gene length (E). **Table S1:** Enrichment of functional categories of uAUG-less and uAUG genes. (PDF 403 KB)

## Abbreviations

CAGE: Cap Analysis of Gene Expression
CDS: Coding sequence
Gro-seq: Global Nuclear Run-On experiment
MEFs: Mouse embryonic fibroblasts
mESCs: Mouse embryonic stem cells
RSAT: Regulatory Sequence Analysis Tools
TE: Translation efficiency
TSS: Transcription start site
uAUG: Upstream AUG
UTR: Un-translated region.
