# Supplementary material for: Co-occurrence of transcription and translation gene regulatory features underlies coordinated mRNA and protein synthesis
Source: BMC Genomics. 2014 Aug 19;15(1):688. doi: 10.1186/1471-2164-15-688 (PMC4158080; doi:10.1186/1471-2164-15-688)
Supplement: Supplementary file 1 — Additional file 1: Figure S1: Translational regulatory features among TATA and TATA-less genes bearing uAUG. A-C. Human and mouse genes containing uAUG in their 5′UTR were grouped according to the presence and absence of a TATA-box in their core promoter region and analyzed for the length of their 5′UTR (A), 3′UTR (B) and coding region (C). The data is presented as boxplots with the median, 25% and 75% quartile values; the top and the bottom whiskers represent the 75–87.5% and 12.5-25% of the population, respectively. In all figures the differences were calculated using the Kruskal-Wallis test and * and *** denote p-value < 0.05 and 0.001, respectively. NS, statistically non significant. The blue and the brown bars represent human and mouse data, respectively. Figure S2: A boxplot presenting the maximal mRNA levels of human uORF-less and uORF genes, retrieved from the SymAtlas v1.2.3. Figure S3: Boxplots presenting the number of exons in human and mouse uORF-less and uORF genes. The blue and the brown bars represent human and mouse data, respectively. Figure S4: Highly mRNA expressing genes are associated with better translational features. A. The prevalence of uAUG in the top 10%, 25% and the bottom 75% mRNA expressing genes. The differences are statistically significant p < 10−4. B-E. Top 25% and bottom 75% mRNA expressing genes, containing or lacking uAUG were analyzed for the length of their 5′UTR (B), 3′UTR (C), coding region (D) and gene length (E). Table S1: Enrichment of functional categories of uAUG-less and uAUG genes. (PDF 403 KB) [file 12864_2014_6399_MOESM1_ESM.pdf]

Fig. S1

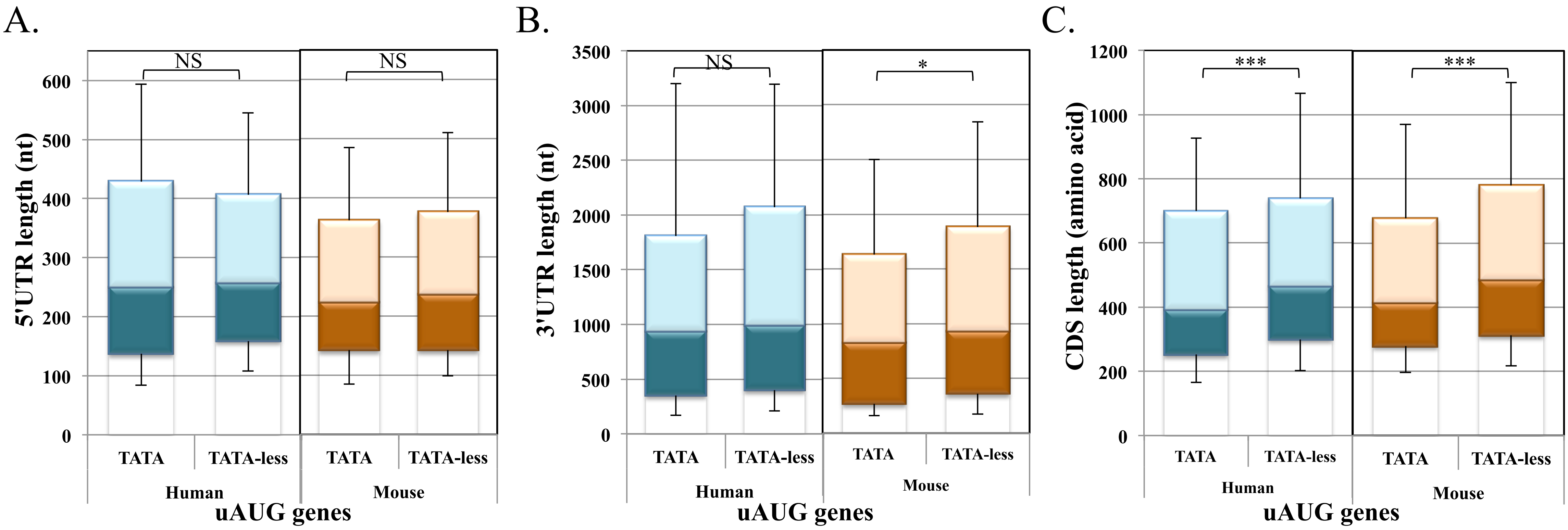

A.
NS
NS
600
500
400
300
200
100
0
5'UTR length (nt)
TATA
TATA-less
TATA
TATA-less
Human
Mouse
uAUG genes
B.
NS
*
3500
3000
2500
2000
1500
1000
500
0
3'UTR length (nt)
TATA
TATA-less
TATA
TATA-less
Human
Mouse
uAUG genes
C.
***
***
1200
1000
800
600
400
200
0
CDS length (amino acid)
TATA
TATA-less
TATA
TATA-less
Human
Mouse
uAUG genes

Fig. S2

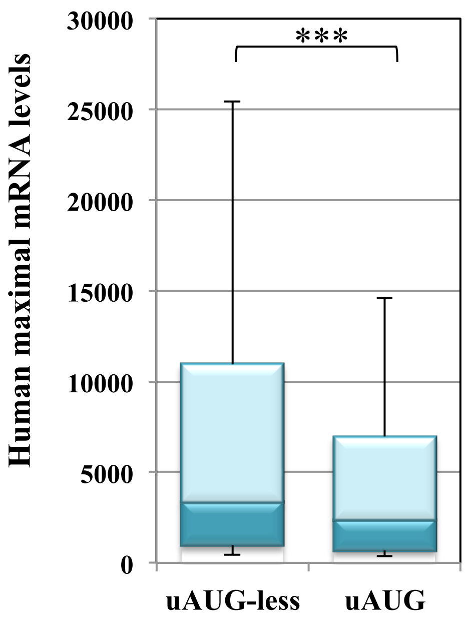
30000
25000
20000
15000
10000
5000
0
Human maximal mRNA levels
***
uAUG-less
uAUG

Fig. S3

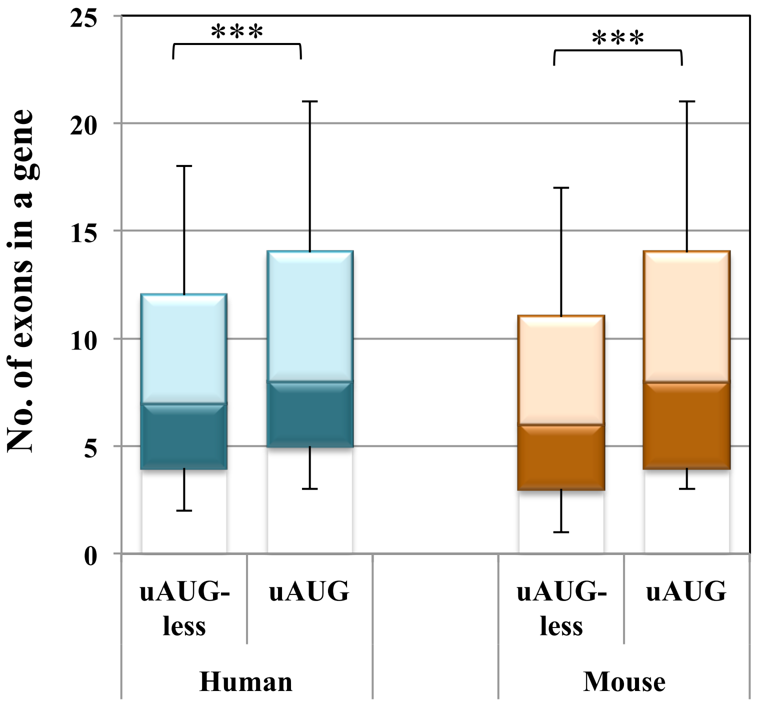
25
20
15
10
5
0
No. of exons in a gene
***
***
uAUG-less
uAUG
uAUG-less
uAUG
Human
Mouse

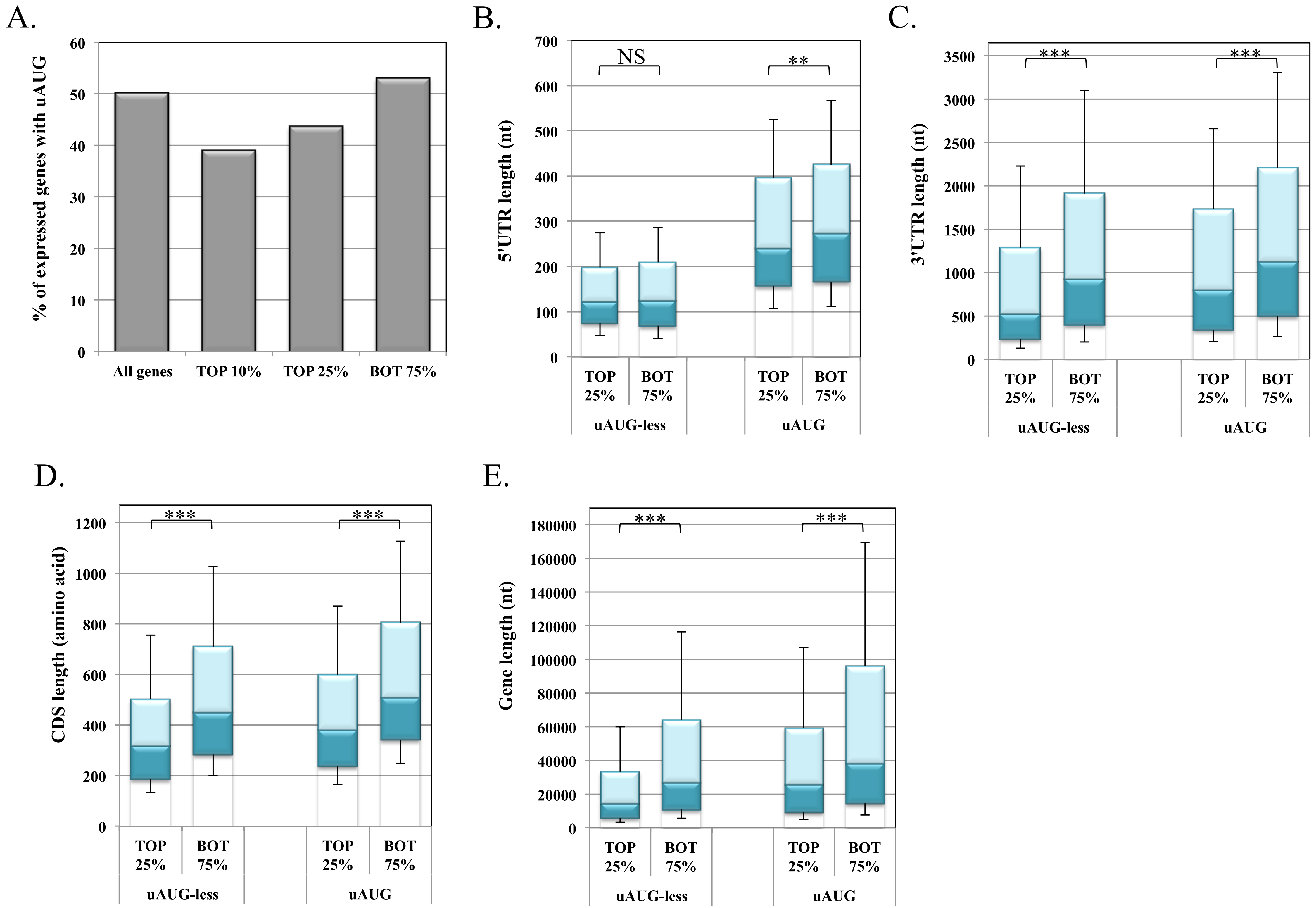
A.
% of expressed genes with uAUG
0
10
20
30
40
50
60
All genes
TOP 10%
TOP 25%
BOT 75%
B.
5'UTR length (nt)
0
100
200
300
400
500
600
700
NS
**
TOP 25%
BOT 75%
uAUG-less
uAUG
C.
3'UTR length (nt)
0
500
1000
1500
2000
2500
3000
3500
***
D.
CDS length (amino acid)
0
200
400
600
800
1000
1200
E.
Gene length (nt)
0
20000
40000
60000
80000
100000
120000
140000
160000
180000

Fig. S4

Table S1

	Term	Enrichment Score	p-value
**uAUG-less**	Olfaction	4.05	1.8E-06
	Signal peptide	3.485	6.1E-03
	Keratin	2.605	1.4E-04
	Nucleosome core	2.1	1.8E-03
**uAUG**	Zinc-finger	4.88	2.7E-06
	Transcription	4.442	3.2E-05
	Plasma membrane part	3.266	1.6E-03
